# Draft Genome Sequence of *Paecilomyces hepiali*, Isolated from *Cordyceps sinensis*

**DOI:** 10.1128/genomeA.00606-16

**Published:** 2016-07-07

**Authors:** Yi Yu, Wenting Wang, Linping Wang, Fang Pang, Lanping Guo, Lai Song, Guiming Liu, Chengqiang Feng

**Affiliations:** a Beijing Key Laboratory of Protection and Utilization of Chinese Medicine, Beijing Normal University, Beijing, China; b CAS Key Laboratory of Genome Sciences and Information, Beijing Institute of Genomics, Chinese Academy of Sciences, Beijing, China; c Resource Center of Chinese Materia Medica, China Academy of Chinese Medical Sciences, Beijing, China

## Abstract

*Paecilomyces hepiali* is an endoparasitic fungus that commonly exists in the natural *Cordyceps sinensis*. Here, we report the draft genome sequence of *P. hepiali*, which will facilitate the exploitation of medicinal compounds produced by the fungus.

## GENOME ANNOUNCEMENT

*Paecilomyces hepiali* is an endoparasitic fungus that commonly exists in the natural *Cordyceps sinensis* anamorph stage. *C. sinensis* has been described as an exotic medicinal mushroom in traditional Chinese and Tibetan medicine (1). Data from the pure compounds are the most revealing in determining the effects of the fungus (2). The chemical constituents isolated from *C. sinensis* include cordycepin (3′-deoxyadenosine) and its derivatives (3). Many studies have demonstrated that cordycepin has significant pharmacological effects, including antiviral, insecticidal, antibacterial, immunostimulatory, and antitumor effects (2, 4, 5). The chemical profiles for *P. hepiali* mycelia are more similar to those for *C. sinensis* (3). *P. hepiali* contains pharmacologically active components similar to those of the natural *C. sinensis* (6).

*P. hepiali* was isolated from *Cordyceps sinensis* from Yushu, Qinghai Province, China. The genome of *P. hepiali* was sequenced using the Illumina HiSeq 2500 sequencing platform with 5 insert libraries (180 bp, 500 bp, 800 bp, 2 kbp, and 5 kbp), providing about 380-fold coverage of the genome. *De novo* assembly of the reads was performed using the SOAP*denovo*2 (7). The gaps inside scaffolds were closed with GapFiller (8), resulting in 88 scaffolds with an *N*_50_ of 2,350 kbp and 660 contigs with an *N*_50_ of 178 kbp. Finally, a 34.6-Mb draft genome sequence with a 53.4% G+C content was obtained for *P. hepiali*.

Gene structure was predicted using an integrated approach. *De novo*, homologue-based, and RNA sequencing (RNA-seq)-based prediction models were conducted with Augustus, GeneWise, and TopHat, respectively. A total of 11,261 gene models were predicted, with an average length of 16,366,717 bp. tRNAs and rRNAs were predicted using tRNAscan-SE (9) and RNAmmer (10), respectively. The functions of genes were also annotated using COG, KEGG (11), and InterProScan (12). The genome contains 113 tRNAs and 23 rRNAs, representing 0.03% of the genome. A total of 6,083 genes were assigned to Clusters of Orthologous Groups (COGs), and 1,829 coding sequences (CDSs) were annotated into pathways using KAAS (13). The data offer a better understanding of *P. hepiali* biology and will facilitate the exploitation of medicinal compounds produced by the fungus.

### Nucleotide sequence accession numbers.

This whole-genome shotgun project has been deposited at DDBJ/EMBL/GenBank under the accession no. LNDK00000000. The version described in this paper is version LNDK02000000.
